# Casticin induces breast cancer cell apoptosis by inhibiting the expression of forkhead box protein M1

**DOI:** 10.3892/ol.2014.1911

**Published:** 2014-02-25

**Authors:** LI-PING LIU, XIAO-CHENG CAO, FEI LIU, MEI-FANG QUAN, XI-FENG SHENG, KAI-QUN REN

**Affiliations:** 1 The Breast Department, Hunan Province Tumor Hospital, Changsha, Hunan 410013, P.R. China; 2 Medical College, Hunan Normal University, Changsha, Hunan 410013, P.R. China

**Keywords:** forkhead box O3, forkhead box protein M1, casticin, breast cancer, therapeutic targets

## Abstract

Casticin is an active ingredient derived from Fructus Viticis, a traditional Chinese medicine. This study aimed to investigate the role of forkhead box O3 (FOXO3a) in breast cancer cells and examine the regulatory mechanisms of FOXO3a in response to casticin treatment of the cells by ELISA, flow cytometry, small interfering RNA (siRNA) transfection and western blot analysis. Casticin treatment induced apoptosis and reduced the expression of the transcription factor forkhead box protein M1 (FOXM1). In addition, FOXM1 repression induced by casticin treatment was associated with the activation of FOXO3a via increased dephosphorylation. Notably, silencing FOXO3a expression by siRNA-mediated gene knockdown attenuated casticin-mediated apoptosis. Collectively, these findings suggest that FOXO3a is a critical mediator of the inhibitory effects of casticin on apoptosis in breast cancer cells.

## Introduction

Despite significant advances in screening techniques that promote early detection of the disease, breast cancer is the leading cause of cancer-related mortality among women worldwide (1). The known risk factors for breast cancer include family history, Li-Fraumeni syndrome, atypical hyperplasia of the breast, a first full-term pregnancy at an advanced age, early menarche and late menopause (2–4). As these risk factors are not easily modifiable (such as genetic predisposition), other strategies for reducing the risk of breast cancer must be investigated. Although selective estrogen receptor (ER) modulators (such as tamoxifen) are effective against ER-positive breast cancers, these agents are ineffective against ER-negative disease (5,6). Moreover, selective ER modulators have severe side effects, including increased risk of uterine cancer, thromboembolism, cataracts and perimenopausal symptoms (5,6). Therefore, novel agents for the prevention and treatment of human breast cancer, particularly hormone-independent breast cancer, are required. Natural products have attracted increasing attention for the discovery of novel anticancer and therapeutic agents (7).

Casticin is one of the active ingredients derived from Fructus Viticis, the fruit of the traditional Chinese medicine *Vitex trifolia L.* (Verbenaceae family) (8). A number of *in vitro* studies have demonstrated that casticin exhibits anticarcinogenic activity in breast (9), prostate (10), lung (11) and colon (12) cancer. Casticin has also been reported to induce cell death of leukemia cells through the induction of apoptosis or mitotic catastrophe (13). We recently reported casticin-induced apoptosis of cervical cancer (14,15) and hepatocellular carcinoma (16) cells; however, the underlying mechanisms remain unclear.

The forkhead/winged helix box class O (FOXO) transcription factors participate in a variety of cell processes, including cell cycle progression, apoptosis, stress detoxification, DNA repair, glucose metabolism and differentiation (17). In mammals, this family of proteins consists of four members, FOXO1, 3, 4 and 6. These factors are regulated by multiple mechanisms, including phosphorylation. The phosphorylated FOXO proteins bind to 14-3-3 chaperone proteins and are sequestered in the cytoplasm where they are unable to regulate gene expression. When active, FOXOs induce cell cycle arrest and apoptosis, negatively mediating oncogenic signaling and acting as antiproliferative factors. Studies in mammalian cells have identified important FOXO target genes involved in the regulation of forkhead box protein M1 (FOXM1) and its downstream target genes, including survivin, p27^Kip1^ and Bim (18). FOXO3a (also known as FOXO3) has been described by several studies as a cell target for antitumor agents in various types of cancers, including breast cancer (19,20) and chronic myeloid leukemia (21). However, the potential roles of FOXO3a/FOXM1 in casticin-induced apoptosis in breast cancer cells had not yet been investigated. Thus, this study aimed to investigate the role of FOXO3a in breast cancer cells and examine the regulatory mechanisms of FOXO3a in response to casticin treatment.

## Materials and methods

### Drugs and chemical reagents

Casticin was purchased from Chengdu Biopurify Phytochemicals Ltd. (Chengdu, China). Casticin has a molecular weight of 374.3, appears as yellow crystals and has a purity of 98.0%. Casticin was prepared in dimethylsulfoxide (DMSO; Sigma-Aldrich, St. Louis, MO, USA) as a 10-mmol/l stock solution and diluted in medium to the indicated concentration prior to use. Mouse monoclonal antibodies against FOXM1, survivin and β-actin were purchased from Santa Cruz Biotechnology, Inc. (Santa Cruz, CA, USA). Mouse anti-human monoclonal antibodies FOXO3a and phospho-FOXO3a-Thr32 were purchased from Millipore (Bedford, MA, USA). The horseradish peroxidase-conjugated goat anti-mouse secondary antibody was purchased from Santa Cruz Biotechnology, Inc. Lipofectamine™ 2000 was purchased from Invitrogen Life Technologies (Carlsbad, CA, USA). The protease inhibitor cocktail, MTT, and all other chemicals were obtained from Sigma-Aldrich.

### Cell culture

The MDA-MB-231 and MCF-7 cell lines were purchased from the China Centre for Type Culture Collection (Wuhan, China) and were maintained in Dulbecco’s modified Eagle’s medium (DMEM, Invitrogen Life Technologies) supplemented with 10% fetal bovine serum (FBS; Hyclone, Logan, UT, USA), 4 mM glutamine, 100 U/ml penicillin and 100 μg/ml streptomycin. The cells were incubated at 37°C in a humidified atmosphere of 5% CO_2_.

### Histone/DNA ELISA for detecting apoptosis

The cell apoptosis ELISA detection kit (Roche, Palo Alto, CA, USA) was used to detect apoptosis in cells treated with casticin according to the manufacturer’s instructions. Briefly, cells were seeded in 96-well plates at a density of 1×10^4^ cells/well. When cells reached 70–80% confluence, testing agents were added to the culture medium containing 10% FBS. After 48 h of culture, the cytoplasm of the cells that was extracted from the control or treatment groups was transferred to 96-well plates, which were pre-coated with streptavidin and previously incubated with a biotinylated mouse anti-histone monoclonal antibody and peroxidase-tagged mouse anti-human DNA monoclonal antibody for 2 h at room temperature. The absorbance was measured at 405 nm under the EXL-800-type enzyme-linked immunosorbent apparatus (Bio-Tek, Winchester, VA, USA).

### Flow cytometry using propidium iodide (PI) staining

The cells were seeded at a density of 4×10^6^ cells/well in 100 ml culture flasks for 24 h and then treated with various concentrations (0.1, 0.5 and 1.0 μM) of casticin for 48 h. PI staining for DNA content was performed as described previously (22). Briefly, the cells were collected and prepared as a single cell suspension by mechanical blowing with PBS (Hyclone), washed twice with cold PBS, fixed with 700 ml/l alcohol at 4°C for 24 h, stained with PI (Sigma-Aldrich) and cell apoptosis was detected using flow cytometry (FACS420, BD Biosciences, Franklin Lakes, NJ, USA).

### DNA agarose gel electrophoresis

The cells were seeded at a density of 4×10^6^ cells/well in 250 ml culture flasks for 48 h and then treated with DMEM containing various concentrations (0.1, 0.5 and 1.0 μM) of casticin or DMSO and 10% FBS for 24 h. The assay was performed as previously described (22). Briefly, cells were washed twice with PBS and DNA was extracted with Apoptotic DNA Ladder Detection kit (Bodataike Company, Beijing, China) according to the manufacturer’s instructions. Extracted DNA was maintained at 4°C overnight. Subsequently, 8.5 μl of the DNA sample was combined with 1.5 μl of 6× buffer solution (New England Biolabs Inc., Ipswich, MA, USA), electrophoresed on 20 g/l agarose gel containing ethidium bromide (BBI Solutions, Madison, WI, USA) at 40 V, and observed using the DBT-08 gel image analysis system (VWR International Ltd., East Grinstead, UK).

### RNA interference

Control non-specific small interfering RNA (siRNA; 5′-UUCUCCGAACGUGUCACGUdTdT-3′) was purchased from Qiagen, Inc. (Valencia, CA, USA). FOXO3A-targeted siRNA (5′-ACUCCGGGUCCAGCUC CAC-3′) was purchased from Santa Cruz Biotechnology, Inc. The cells were seeded in six-well plates and transfected at 50% confluence with either 200 nmol/l of control non-specific siRNA or FOXO3a-specific siRNA using Oligofectamine™ reagent (Invitrogen Life Technologies) according to the manufacturer’s instructions. After 24 h of transfection, the cells were treated with DMSO (control) or 0.5 μM casticin for 48 h. The cells were then collected and processed for western blotting and histone/DNA ELISA.

### Western blot analysis

The cells (1×10^6^) were seeded in 100-mm culture dishes, allowed to attach by overnight incubation and treated with DMSO (control) or 0.5 μM casticin for the specified time periods. Cell lysates were prepared as previously described (22). Lysates were cleared by centrifugation at 16,873 × g for 30 min. Lysate proteins were resolved by 10 or 12.5% SDS-PAGE (Millipore) and transferred to polyvinylidene fluoride membranes. The membranes were incubated with Tris-buffered saline containing 0.05% Tween 20 and 5% (w/v) non-fat dry milk. The membranes were then treated with the desired primary antibody for 1 h at room temperature or overnight at 4°C. Following treatment with the horseradish peroxidase-conjugated goat anti-mouse secondary antibody the immunoreactive bands were visualized using an enhanced chemiluminescence kit (Amersham Pharmacia Biotech, Piscataway, USA). The blots were stripped and re-probed with anti-actin antibody to normalize for differences in protein loading. Changes in the level of the desired protein were determined by densitometric scanning of the immunoreactive band and corrected for the β-actin loading control. Immunoblotting for each protein was performed at least twice using independently prepared lysates to ensure reproducibility of the results.

### Statistical analysis

The data were analyzed using SPSS software, version 15.0 (SPSS Inc., Chicago, IL, USA). Data are expressed as the means ± standard deviation. The means of multiple groups were compared with one-way analysis of variance, after the equal check of variance, and the comparisons among the means were performed using the least significant difference method. Statistical comparison was also performed with Dunnett’s two-tailed t-test when appropriate. P<0.05 was considered to indicate a statistically significant difference.

## Results

### Effects of casticin on breast cancer cell apoptosis

It has previously been reported that casticin inhibits the growth of MCF-7 human breast cancer cells and induces G2/M cell cycle arrest (9). Thus, whether casticin exerts any effect on apoptosis of the estrogen-responsive MCF-7 or the estrogen-independent MDA-MB-231 breast cancer cell lines was investigated. ER-positive MCF-7 cells were originally isolated from pleural effusion of a stage IV invasive ductal carcinoma. These cells are aneuploid with high chromosomal instability and are defective for the G1 and mitotic spindle checkpoints. However, the cells express wild-type p53 (23). The MDA-MB-231 cell line, which was derived from a stage IV invasive ductal carcinoma, is ER-negative, partially proficient for all cell cycle checkpoints and expresses mutant p53 (23).

**Figure 1:**
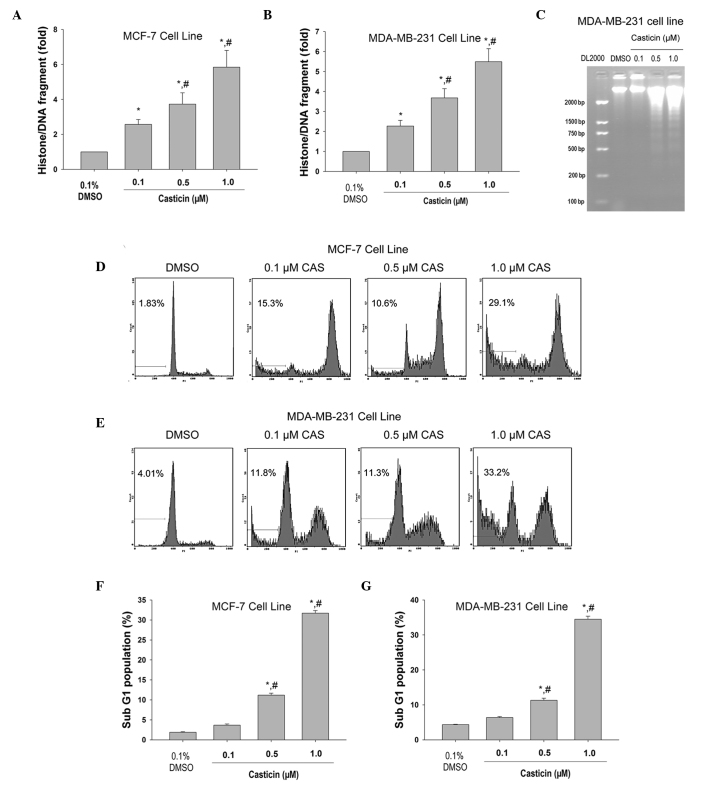
Induction of apoptosis by casticin in breast cancer cells. Cells were treated with casticin at the indicated concentrations for 48 h. Increase of histone/DNA fragment levels by casticin in (A) MCF-7 and (B) MDA-MB-231 cell lines was observed. (C) Following treatment of MDA-MB-231 cells for 48 h, fragmented DNA was extracted from the treated cells and analyzed on a 2.0% agarose gel. Promotion of sub-G1 population by casticin in (D and F) MCF-7 and (E and G) MDA-MB-231 cell lines was observed. Data and error bars are presented as means ± SD. ^*^P<0.05, vs. treatment with DMSO and ^#^P<.05, vs. treatment with 0.1 μM casticin. DMSO, dimethylsulfoxide; cas, casticin.

After 48 h of exposure, casticin significantly induced histone/DNA fragmentation in a concentration-dependent manner in MCF-7 (Fig. 1A) and MDA-MB-231 cells (Fig. 1B). Agarose gel electrophoresis revealed a typical ladder pattern of internucleosomal DNA fragmentation in MDA-MB-231 cells treated with 0.5 and 1.0 μM casticin (Fig. 1C). Flow cytometry analysis showed that casticin treatment resulted in increased sub-G1 population in MCF-7 (Fig. 1D and F) and MDA-MB-231 (Fig. 1E and G) cells (P<0.05) in a concentration-dependent manner. Overall, these findings suggest that casticin induces breast cancer cell apoptosis.

### Effects of casticin on the expression of FOXM1 in breast cancer cells

**Figure 2:**
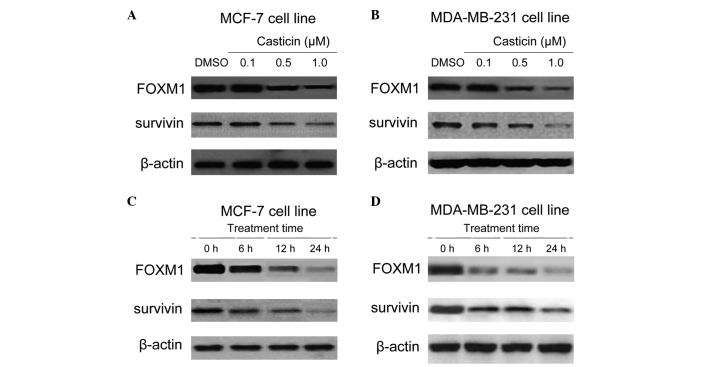
Western blot analysis revealing a reduction of FOXM1 and survivin protein expression by casticin in breast cancer cells. (A) MCF-7 and (B) MDA-MB-231 cells were treated with casticin at the indicated concentrations for 24 h. (C) MCF-7 and (D) MDA-MB-231 cells were treated with 0.5 μM casticin for the indicated times. β-actin was used as a loading control. FOXM1, forkhead box protein M1; DMSO, dimethylsulfoxide.

Previous research, including a study by Wang *et al* (24), has demonstrated that FOXM1 is a novel target of natural active compounds (24,25). Thus, whether FOXM1 is a downstream signaling target of casticin in breast cancer cells was investigated. Dose titration of casticin in the MCF-7 and MDA-MB-231 cell lines was performed, and the effects on FOXM1 and its downstream target survivin were assayed (Fig. 2A and B). A dose of 0.5 μM was selected for subsequent experiments. The MCF-7 and MDA-MB-231 cells were then treated with 0.5 μM casticin for 0, 6, 12 and 24 h. Western blot analysis revealed that casticin treatment decreased FOXM1 expression and this coincided with a decrease in the FOXM1 target, survivin (Fig. 2C and D). Collectively, these findings suggest that FOXM1 is a cellular target of casticin in breast cancer cells.

### Effects of casticin on the phosphorylation of FOXO3a in breast cancer cells

**Figure 3:**
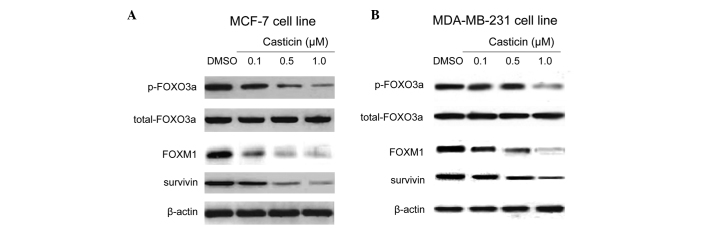
Reduction of FOXO3a phosphorylated protein expression by casticin in breast cancer cells. (A) MCF-7 and (B) MDA-MB-231 cells were treated with casticin at the indicated concentrations for 24 h. The expression of p-FOXO3a, total-FOXO3a, FOXM1 and survivin proteins were analyzed by western blotting. β-actin was used as a loading control. FOXM1, forkhead box protein M1; FOXO3a, forkhead box O3; p-FOXO3a, phosphorylated FOXO3a.

FOXO3a is an upstream regulator of FOXM1. Additionally, the antiproliferative and apoptotic effects of genistein, an isoflavone derived from soybeans, were partly mediated through the regulation of Akt/FOXO3a signaling (26). Thus, phosphorylated FOXO3a protein was examined in order to determine whether differences in the expression or activity of signaling regulators may enhance the effect of casticin on FOXM1. Western blot analysis revealed that treatment with casticin led to a decrease in FOXO3a phosphorylation and a corresponding reduction in FOXM1 and its target, survivin (Fig. 3A and B). These findings suggest that the casticin-induced repression of FOXM1 may be associated with FOXO3a activation.

### Effects of FOXO3a silencing on casticin-mediated apoptosis of breast cancer cells

**Figure 4:**
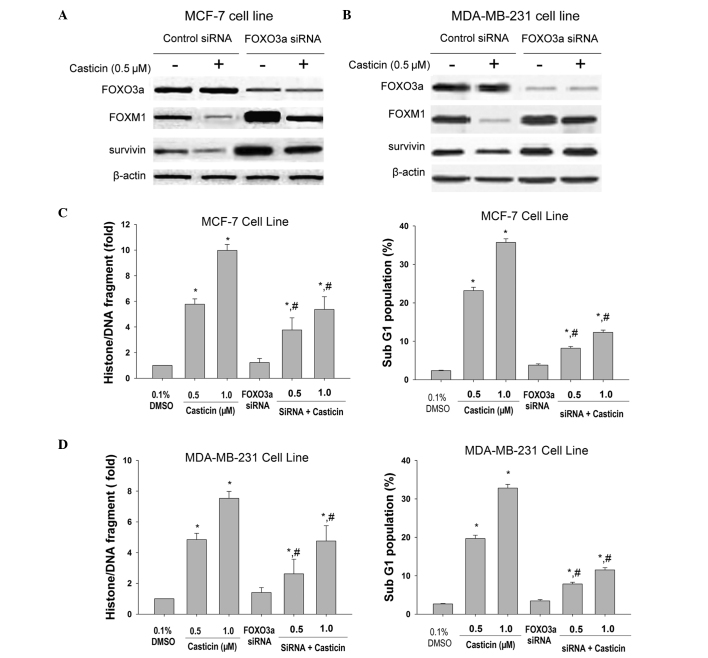
Depletion of FOXO3a by siRNA increases FOXM1 and survivin protein expression and attenuates the apoptotic effects of casticin in breast cancer cells. Cells were transiently transfected with a control non-specific siRNA or a FOXO3a-targeted siRNA for 24 h, followed by treatment with DMSO or 0.5 μM casticin for 24 h. The expression of FOXO3a, FOXM1 and survivin proteins in (A) MCF-7 and (B) MDA-MB-231 cell lines were analyzed using western blotting. β-actin was used as a loading control. ELISA and flow cytometry revealed an induction of histone/DNA fragment levels and an increased sub-G1 population, respectively in the (C) MCF-7 and (D) MDA-MB-231 cell lines. Data and error bars are presented as means ± SD. ^*^P<0.05, vs. treatment with DMSO in cells transfected with the non-specific siRNA and ^#^P<0.05, vs. treatment with casticin at the same concentrations in cells transfected with the non-specific siRNA. FOXO3a, forkhead box O3; siRNA, small interfering RNA; FOXM1, forkhead box protein M1; DMSO, dimethylsulfoxide.

In order to determine the importance of FOXO3a in the cellular response to casticin, the MCF-7 and MDA-MB-231 cells, which express high protein levels of FOXO3a, were transfected with specific siRNAs. As shown in Fig. 4A and B, FOXM1 and survivin proteins were increased in FOXO3a-knockdown cells. The decrease of FOXO3a significantly attenuated the apoptotic effects of casticin in breast cancer cells (Fig. 4C and D). These findings support the hypothesis that casticin induces breast cancer cell apoptosis by inducing FOXO3a activity, which represses FOXM1.

## Discussion

This study demonstrated that the polymethoxyflavone compound, casticin, induces apoptosis through the activation of FOXO3a. This correlates with casticin-mediated inhibition of FOXM1 and survivin, which are downstream targets of FOXO3a. Inhibition of FOXO3a by siRNA predominantly blocks casticin-induced apoptosis. Previous studies have demonstrated the antiproliferative and pro-apoptotic effects of casticin in prostate (10), cervical (14,15), lung (11) and colon (12) cancer. This study investigated the role of FOXO transcription factors in mediating the effects of casticin. As casticin is a non-toxic polyphenolic compound, it is safe to use for the treatment and/or prevention of breast cancer.

In the present study, the role and regulation of FOXM1 in response to casticin treatment in breast cancer cells was investigated. Our findings demonstrated that casticin repressed the expression of FOXM1 in breast cancer cells, which was associated with the downregulation of FOXM1 activity, revealed by the concomitant decrease in expression of its downstream target, survivin. As casticin targets FOXM1 through FOXO3a in breast cancer, it is possible to increase the efficacy of casticin by targeting FOXM1. FOXM1 has been reported as a valid target for the development of anticancer therapeutics (17). For example, a novel thiazole antibiotic, thiostrepton, selectively induced cell cycle arrest and cell death in breast cancer cells through the downregulation of FOXM1 expression (26). Similarly, other native compounds, such as resveratrol and genistein, have also been found to repress the expression of FOXM1 and cell proliferation (26–28). Furthermore, a cell-permeable ARF peptide inhibitor of FOXM1 has been shown to selectively induce apoptosis in human hepatocellular carcinoma cell lines and mouse models (29).

FOXO transcription factors play important roles in the regulation of apoptosis (30). In the present study, FOXO3a was key in the regulation of the anti-apoptotic gene, survivin. In accordance with our findings, FOXO silencing has been shown to decrease the expression levels of Bim, TNF-related apoptosis-inducing ligand, Fas ligand (FasL) and p27^Kip1^, which are all FOXO target genes controlling the cell cycle and apoptosis (31–33). Inhibition of the PI3K/Akt and MEK/ERK pathways act synergistically to regulate the anti-angiogenic effects of epigallocatechin gallate, resveratrol and sulforaphane through activation of FOXO transcription factors (25,34,35). The FOXO transcription factors regulate tissue homeostasis in the pancreas and in individuals with diabetes and cancer. FOXO regulates apoptotic genes, such as Bim, FasL and survivin (36). Collectively, those findings suggest that activation of FOXO transcription factors by chemopreventive agents may regulate apoptosis. Akt and ERK have been shown to directly phosphorylate and inactivate FOXO transcription factors resulting in cytoplasmic retention, inactivation and inhibition of the expression of FOXO-regulated genes. This enables the control of various cell processes, such as metabolism, cell cycle, cell death and oxidative stress (37). Our findings suggested that casticin inhibits the cytoplasmic phosphorylation of FOXO3a. Depletion of FOXO3a levels by siRNA abrogates casticin-induced apoptosis. Overall, these results demonstrate that the activation of FOXOs has significant implications for the treatment and prevention of breast cancer.

Notably, the MDA-MB-231 triple-negative breast cancer (TNBC) cell line was sensitive to casticin treatment. TNBC is clinically characterized as more aggressive and less responsive to standard treatments. Searching for effective strategies for the treatment of TNBC has become a high priority in breast cancer therapy. Our results warrant further investigation to determine whether casticin may serve as a novel candidate agent for the management of TNBC. Identification of casticin as a potent anti-TNBC agent may have a significant effect on developing novel therapeutic strategies for the treatment of TNBC.

In summary, our study suggests that FOXO3a/FOXM1/survivin are cellular targets and markers of casticin action in breast cancer. Furthermore, FOXM1 functions downstream of FOXO3a in response to casticin. These findings may have important implications for the development of therapeutic agents for breast cancer.
